# Supplementary material for: Single-Walled Carbon Nanohorns as Boosting Surface for the Analysis of Low-Molecular-Weight Compounds by SALDI-MS
Source: Int J Mol Sci. 2022 Apr 30;23(9):5027. doi: 10.3390/ijms23095027 (PMC9100638; doi:10.3390/ijms23095027)
Supplement: Supplementary file 1 [file ijms-23-05027-s001.zip › ijms-1672079-supplementary.pdf]

Single-walled carbon nanohorns as boosting surface for the analysis of low-molecular weight compounds by SALDI-MS

Marco Roverso[1*], Roberta Seraglia[2], Raghav Dogra[1], Denis Badocco[1], Silvia Pettenuzzo[1,3], Luca Cappellin[1], Paolo Pastore[1], Sara Bogialli[1].

1. Department of Chemical Sciences, University of Padova, Padova, Italy
2. Institute of Condensed Matter Chemistry and Technologies for Energy (ICMATE), National Research Council-CNR, Padova, Italy
3. Center Agriculture Food Environment (C3A), University of Trento, San Michele all'Adige, Italy

Corresponding Author:

Marco Roverso

Department of Chemical Sciences, University of Padova

Via Marzolo, 1

IT35131 Padova, Italy

marco.roverso@unipd.it

Phone: +39 049 8275181

Supplementary Material

Table S1: Ionic species identified in the analysis of a water solution of glycine and phenylalanine (1 mg/mL each) by SALDI-MS. Spectra reported in Figure 2. Mass accuracy: <150 ppm.

Detected Species	Glycine (Figure 2, panel A)	Phenylalanine (Figure 2, panel B)
$[M+H]^+$	Not detected	166.090
$[M+Na]^+$	98.023	188.085
$[M+K]^+$	114.009	204.075
$[M-H+2Na]^+$	120.005	210.055
$[M-H+Na+K]^+$	135.984	226.033
$[M-H+2K]^+$	151.962	242.007

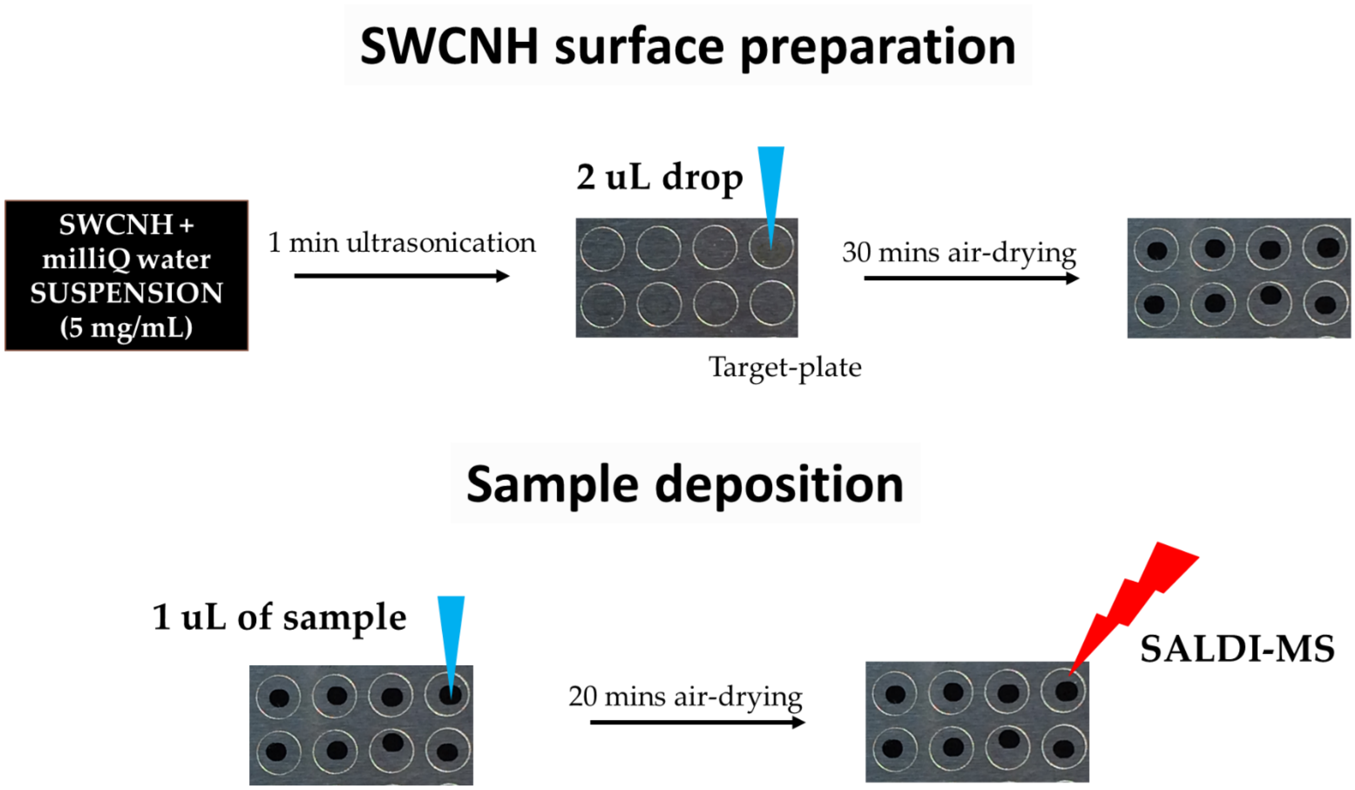

Figure S1: Schematic representation of the optimized procedure for the preparation of the SWCNH-based surface and sample deposition.
